# Broadband dielectric spectroscopy for monitoring temperature-dependent chloride ion motion in BiOCl plates

**DOI:** 10.1038/s41598-020-79018-2

**Published:** 2020-12-16

**Authors:** Adrian Radoń, Dariusz Łukowiec, Patryk Włodarczyk

**Affiliations:** 1 Łukasiewicz Research Network - Institute of Non-Ferrous Metals, Sowinskiego 5 St., 44-100 Gliwice, Poland; 2 Faculty of Mechanical Engineering, Silesian University of Technology, Konarskiego 18 a St., 44-100 Gliwice, Poland

**Keywords:** Materials for devices, Materials for energy and catalysis, Applied physics, Electronics, photonics and device physics

## Abstract

The dielectric properties and electrical conduction mechanism of bismuth oxychloride (BiOCl) plates synthesized using chloramine-T as the chloride ion source were investigated. Thermally-activated structure rebuilding was monitored using broadband dielectric spectroscopy, which showed that the onset temperature of this process was 283 K. This rebuilding was related to the introduction of free chloride ions into [Bi_2_O_2_]^2+^ layers and their growth, which increased the intensity of the (101) diffraction peak. The electrical conductivity and dielectric permittivity were related to the movement of chloride ions between plates (in the low-frequency region), the interplanar motion of Cl^−^ ions at higher frequencies, vibrations of these ions, and charge carrier hopping at frequencies above 10 kHz. The influence of the free chloride ion concentration on the electrical conductivity was also described. Structure rebuilding was associated with a lower concentration of free chloride ions, which significantly decreased the conductivity. According to the analysis, the BiOCl plate conductivity was related to the movement of Cl^−^ ions, not electrons.

## Introduction

Oxychlorides have unique properties due to their structure anisotropy. Many different oxychlorides, such as BiOCl, CaOCl_2_, FeOCl, CuOCl, and ZnOC, have recently been synthesized and tested, especially for use as photocatalysts^1–4^. Metal oxychloride materials have been investigated because of their chemical stability and their ability to form 2D and 3D nanostructures^2,5,6^. Interest in the nanostructures of bismuth oxychloride BiOCl is related to their unique optical and electrical properties. Bismuth oxychloride is a semiconductor with a matlockite-type structure with Cl^−^ ions located between [Bi_2_O_2_]^2+^ layers. This structure is responsible for the unique optical, electrical, and catalytic properties of this material. It was confirmed that this charged layered structure can create internal dipole moments^7^, which is related to the strong intralayer covalent bonding of [Bi_2_O_2_]^2+^ and weak interlayer van der Waals interactions of Cl^−^ double slabs. Moreover, it is possible to create vacancies in the oxychloride structure^8,9^, which appear in the dielectric spectra and are responsible for the high photocatalytic activity of these materials. BiOCl has been used as a catalyst, especially for the photocatalytic degradation of organic compounds. The influence of shape on the photocatalytic activity of BiOCl for the degradation of toxic bisphenol A was tested for microplates and microflowers, and it was confirmed that the higher adsorption capacity and presence of more active sites in the microflowers enhanced their photocatalytic activity^10^. Other BiOCl-based nanostructures such as BiOCl/Bi_2_S_3_, BiOCl/(BiO)_2_CO_3_, and BiOCl/g-C_3_N_4_ heterostructures are also good photocatalysts^11–13^.

Due to their potential applications, especially in chloride ion batteries, gas sensors, and supercapacitors, it is important to understand the dielectric properties and electrical conduction mechanism of BiOCl structures over a broad frequency range^14–19^. The electrical conductivity of BiOCl nanosheets as a function of temperature and oxygen partial pressure was described by Myung et al.^20^ Their results showed that the band gap of BiOCl nanosheets was 3.34 eV, with a conduction band edge of − 3.36 eV and a valence band edge of − 6.97 eV. They also calculated that the activation energy of conduction below 1000 mbar of oxygen from 300 to 425 K was 862 meV; however, there have been no systematic studies that describe the dielectric properties of BiOCl. Most research has focused on determining its optical and electrical properties, including charge transport in the UV–Vis range^8,21–23^; therefore, the main goal of recent studies has focused on vacancies and hole-electron generation. Determining charge carrier motion at lower frequencies can broaden the possible applications of oxychloride structures, especially for gas sensors. For example, BiOCl was tested as a gas sensor for CO, CO_2_, and O_2_ at 100 kHz by Michel et al.^14^ who showed that the sensing ability of the sensor was related to electron transport from BiOCl to adsorbed O_2_. According to this, the number of holes increased in BiOCl, which increased the conductivity. Describing charge carrier transport in BiOCl plates should also clarify the sensing properties at lower frequencies and help correlate the dielectric properties with gas sensing. Describing charge carrier motion in oxychlorides should also extend research on the use of oxychlorides as electrodes, especially in chloride ion batteries.

This work focused on describing the dielectric properties and electrical conduction mechanism of BiOCl plates synthesized using chloramine-T as the chloride ion source. Moreover, the influence of structure rebuilding on the structure, conductivity, and chloride ion motion was described using a combination of X-ray diffraction, transmission electron microscopy, and dielectric spectroscopy over broad temperature (173–373 K) and frequency (0.05 Hz–1 MHz) ranges.

## Experimental section

### Synthesis of BiOCl plates

10 g of chloramine-T (CH_3_C_6_H_4_SO_2_NClNa·3H_2_O) was dissolved in 100 ml of deionized (DI) water. Separately, a solution containing 20 mmol of Bi(NO_3_)_3_·5H_2_O in 50 ml of DI water was prepared. The Bi^3+^ solution was added into the chloramine-T solution, together with 5 ml of HNO_3_ (65%). The obtained suspension was heated at reflux for 1 h. Afterward, the suspension was cooled to room temperature and a white precipitated was collected by filtration using quantitative filter paper and then washed with DI water, ethanol, and acetone to remove impurities and water. The synthesized BiOCl was dried at room temperature for 24 h.

### Characterization of materials

The structure rebuilding of BiOCl plates, as well as the structure of as-synthesized plates, were examined using scanning transmission electron microscopy (STEM) and transmission electron microscopy (TEM). The BiOCl dispersion before and after heat treatment was prepared by sonicating a few milligrams of the materials in ultrapure ethanol. The measurements were performed in scanning and transmission modes using an S/TEM TITAN 80-300 according the procedure described before^24^. The crystal structure of the BiOCl plates was determined using X-ray diffraction (XRD). Powder XRD patterns were recorded using a Rigaku MiniFlex 600 X-ray diffractometer with a copper tube, Cu Kα (*λ* = 0.15406 nm), a tube voltage of 40 kV, and a current of 15 mA, using a D/teX Ultra silicon strip detector^24^. The dielectric properties and electrical conduction mechanism were determined using a Concept 81 dielectric spectrometer equipped with an Alpha analyser and a Novo-cool temperature control system. Complex dielectric permittivity and complex conductivity were measured for compressed (pressure = 30 bar) samples in the form of plates with a diameter of 10 mm and a thickness of 1–1.5 mm over a broad frequency range from 0.05 Hz to 1 MHz. To determine the influence of temperature on the dielectric properties, structure rebuilding measurements were conducted in a wide temperature range (173–373 K) with Δ*T* = 10 K. This procedure was applied in previous works to determine properties of different materials such as ferrites and composites^24–26^.

## Results and discussion

### Structure and morphology

**Figure 1:**
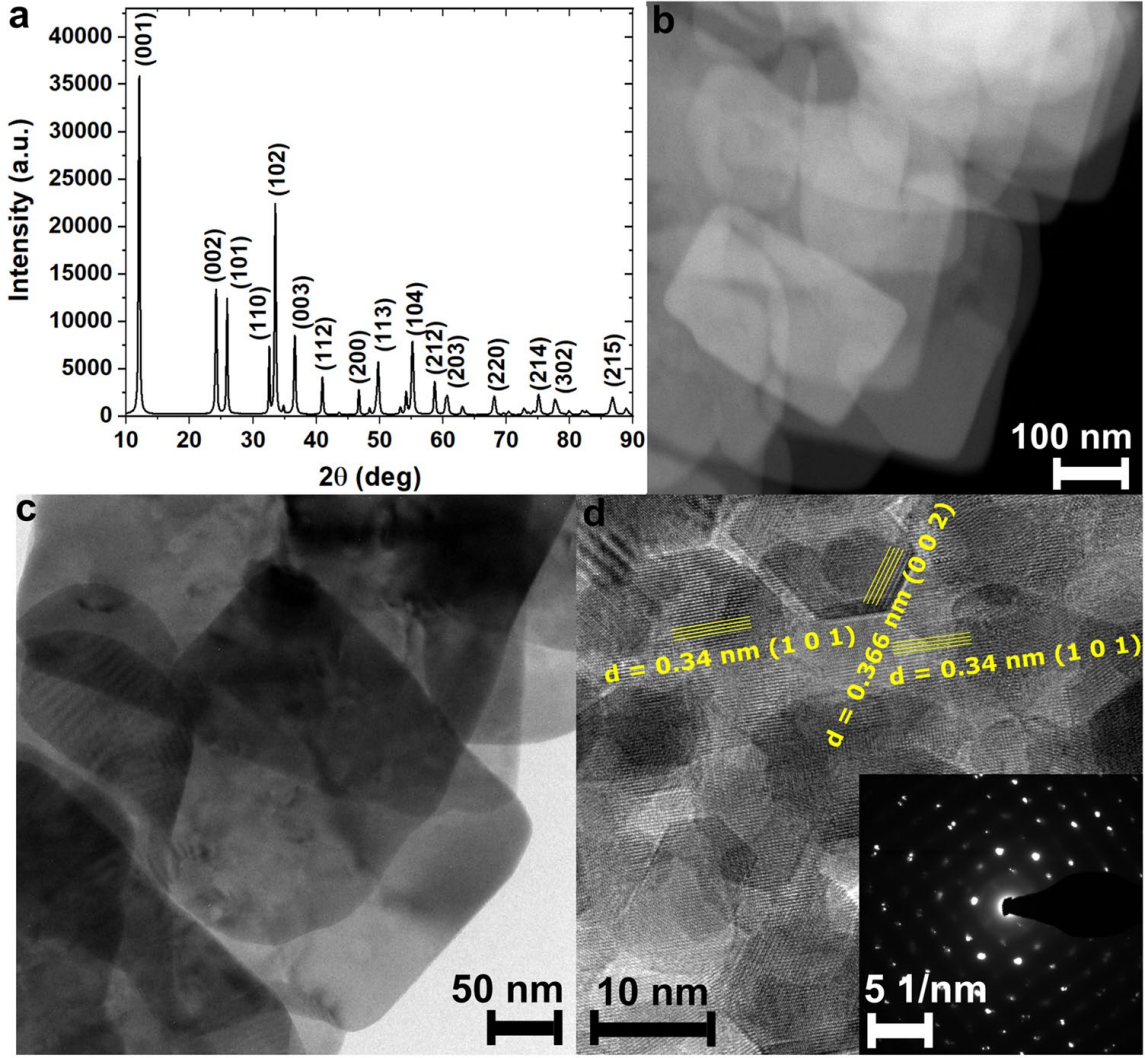
Structure and morphology of BiOCl plates: (**a**) XRD pattern of as-synthesized BiOCl using chloramine-T with marked Miller indices for peaks with the highest intensities; (**b**) HAADF STEM and (**c**) BF-DF STEM images of BiOCl; (**d**) TEM image of BiOCl plate with marked planes characteristic of BiOCl; inset shows the SEAD pattern.

The structure and phase purity of as-synthesized BiOCl were examined using XRD. The collected XRD patterns with marked Miller indices (129: P4/nm; DB card number: 1011175) are presented in Fig. 1a. The analysis confirmed that the sample was pure. The high-intensity (001) peak was related to the layered structure, in which covalently-bonded [Bi_2_O_2_]^2+^ layers were separated by chloride ions. To visualize the morphology and structure of BiOCl synthesized using chloramine-T, the STEM and TEM images were analysed. Organic modifiers, chloride ion sources, and reaction conditions (time, temperature, and solvents) play crucial roles in the formation of BiOCl structures with different morphologies^27,28^. The influence of chloride ion sources on the shape of BiOCl structures was described by Zhao et al.^29^ Three different types of nanoplate-assembled BiOCl microflowers were synthesized using NaCl, choline chloride, imidazole hydrochloride, and chlorinated pyridine. The representative STEM and TEM images of as-synthesized BiOCl are presented in Fig. 1b–d. As can be seen in Fig. 1b,c , the synthesized material was composed of stacked plates, and the TEM image in Fig. 1d confirms that these plates are composed of many small crystallites with visible boundaries.

### Dielectric properties

To determine dielectric properties and electrical conduction mechanism, dielectric spectra of compressed samples were recorded.

**Figure 2:**
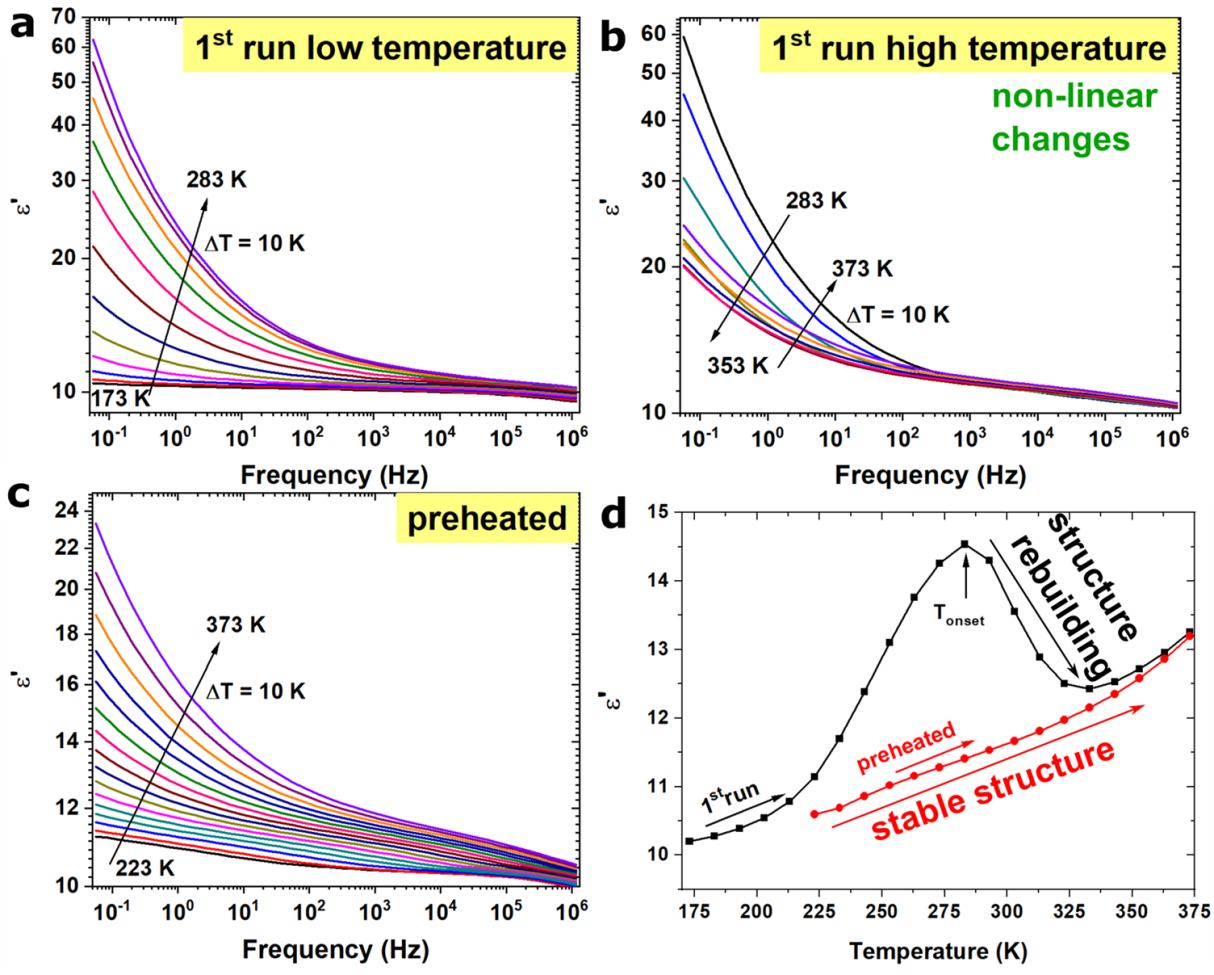
Dielectric spectra of BiOCl plates: (**a**) real part of complex permittivity as a function of temperature measured for as-synthesized, compressed sample; (**b**) real part of complex permittivity as a function of temperature measured in the high-temperature region, with marked nonlinear changes; (**c**) real part of complex permittivity as a function of temperature measured for preheated BiOCl plates; (**d**) influence of temperature on the real part of complex permittivity plotted for the sample before (1st run) and after heat treatment (2nd run), with the temperature of the thermally-activated process marked.

As can be seen in Fig. 2a. the value of ε′ increases with increasing temperature up to 283 K. These changes are much more visible in the low-frequency region, which may be related to the space charge polarization at grain boundaries and the accumulation of free charge carriers at the interface between the sample and copper electrode. This behavior is natural and has been observed for various materials, such as ferrites and perovskites^25,30^. At increasing frequencies, ε′ decreases, which is related to the limited charge carrier movement and the absence of grain boundary polarization; however, during continuous heating up to 373 K, nonlinear changes can be observed (Fig. 2b). After reaching the highest ε′ value at 283 K, the real part of complex permittivity decreases with increasing temperature up to 353 K and then increases again with temperature. This unexpected behavior is likely related to thermally-generated changes in the sample structures. These changes influence the polarization of grain boundaries and the accumulation of free charge carriers. To check the reversibility of this process, the same sample was measured again from 223 to 373 K (sample marked as preheated). As Fig. 2c show, ε′ increases successively with increasing temperature; however, it never reaches the same level as the first measurement, even at higher temperatures. The observed changes can be also presented as ε′ (T) plots for the 1st and 2nd runs (before and after heating) at a constant frequency of 100 Hz (Fig. 2d). During the first heating, a thermally-activated process at *T*_*onset*_ = 283 K can be observed. This nonreversible process is likely related to changes in the number of charge carriers.

**Figure 3:**
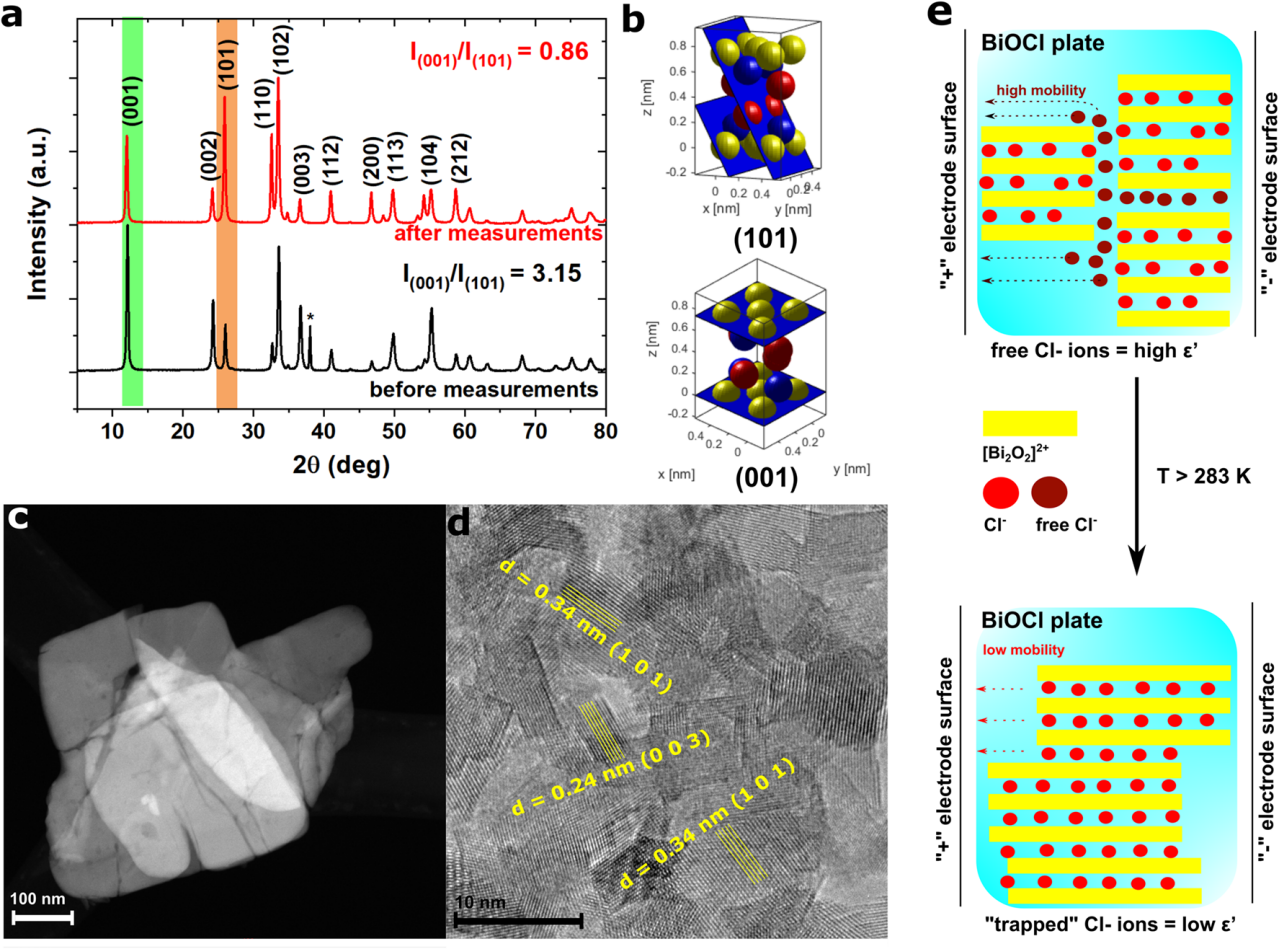
(**a**) Comparison of XRD patterns of the same BiOCl sample before and after measurements, confirming structure rebuilding [the (*) peak represents the X-ray specimen holder]; (**b**) visualization of two characteristic planes in the BiOCl unit cell marked on (a): yellow balls represent Bi^3+^ ions, blue ones O^2−^, and red ones Cl^−^ ions (CrysTBox cellViewer 1.13^31,32^); (**c**) HAADF STEM image of BiOCl plates after compression and heat treatment; (**d**) TEM image of BiOCl plate after compression and heat treatment with marked planes characteristic of BiOCl; (**e**) schematic representation of structure rebuilding and influence of this process on chloride ion mobility.

To confirm this, measurements were repeated for the same compressed sample measured before and after heating. The XRD patterns of these two BiOCl samples (after compressing) are presented in Fig. 3, which shows changes in the intensity of the peaks. For example, the ratio between the intensity of (001) and (101) peaks decreased from 3.15 to 0.86 after heat treatment (Fig. 3a). According to this, the observed nonlinear changes in the dielectric spectra are related to the structure rebuilding.

The changes in the structure and morphology after heat treatment were presented in the HEEADF STEM and TEM images (Fig. 3c,d). The HAADF STEM image clearly shows that the preheated BiOCl after compression also has a plate-like structure, but some cracks were visible. Therefore any changes generated by the heat treatment are a result of the growth of the crystallites. In the sample before heat treatment, the preferred orientation is a (001) plane in which chloride ions exist between [Bi_2_O_2_]^2+^ layers. These chloride ions are trapped and connected with layers only by van der Waals interactions, therefore they are characterized by the high mobility and can accumulate as free Cl^−^ ions on grain boundaries (Fig. 3e). After heat treatment the preferred orientation is a (101) plane. As can be seen in Fig. 3b, for this plane to be preferred, chloride ions must be incorporated between the [Bi_2_O_2_]^2+^ layers. In order for this process to take place, the rebuilding of the structure has to occur. As presented in Fig. 1d, the as-synthesized BiOCl plates were composed of ultrafine crystallites with a well-formed crystalline structure. After heat treatment, structure rebuilding was also observed in the TEM image. As can be seen in Fig. 3d, the boundaries between well-formed crystallites have disappeared and a homogenous structure was formed. According to that, the increasing intensity of the (101) diffraction peak is related to the incorporation of free chloride ions into positively charged layers, whereas the number of these layers is nearly constant.

The mobility of chloride ions is increasing with temperature up to 283 K, at which structure rebuilding occurs. Highly mobile Cl^−^ ions can be “trapped” between [Bi_2_O_2_]^2+^ layers, which changes the crystal structure upon further heating the sample. This process was not observed during the second run, which was associated with the presence of a stable structure in which free Cl^−^ ions did not occur (Fig. 3e).

The second useful parameter for describing the motion of charge carriers in inorganic materials is the complex electric modulus, especially its imaginary part. This parameter can describe the transition between the conductivity in grains and by grain boundaries. The complex electric modulus can be expressed as:

1 $$ M^{*} = M^{{\prime }} + iM^{{\prime \prime }} = \frac{{ \varepsilon^{{\prime }} }}{{\left| { \varepsilon^{*} } \right|^{2} }} + \frac{{i \varepsilon^{{\prime \prime }} }}{{\left| { \varepsilon^{*} } \right|^{2} }} $$

where *M*′ and *M″* are the real and imaginary parts of the complex electric modulus M^*^, ε′ and *ε″* are the real and imaginary parts of the complex permittivity ε^*^.

**Figure 4:**
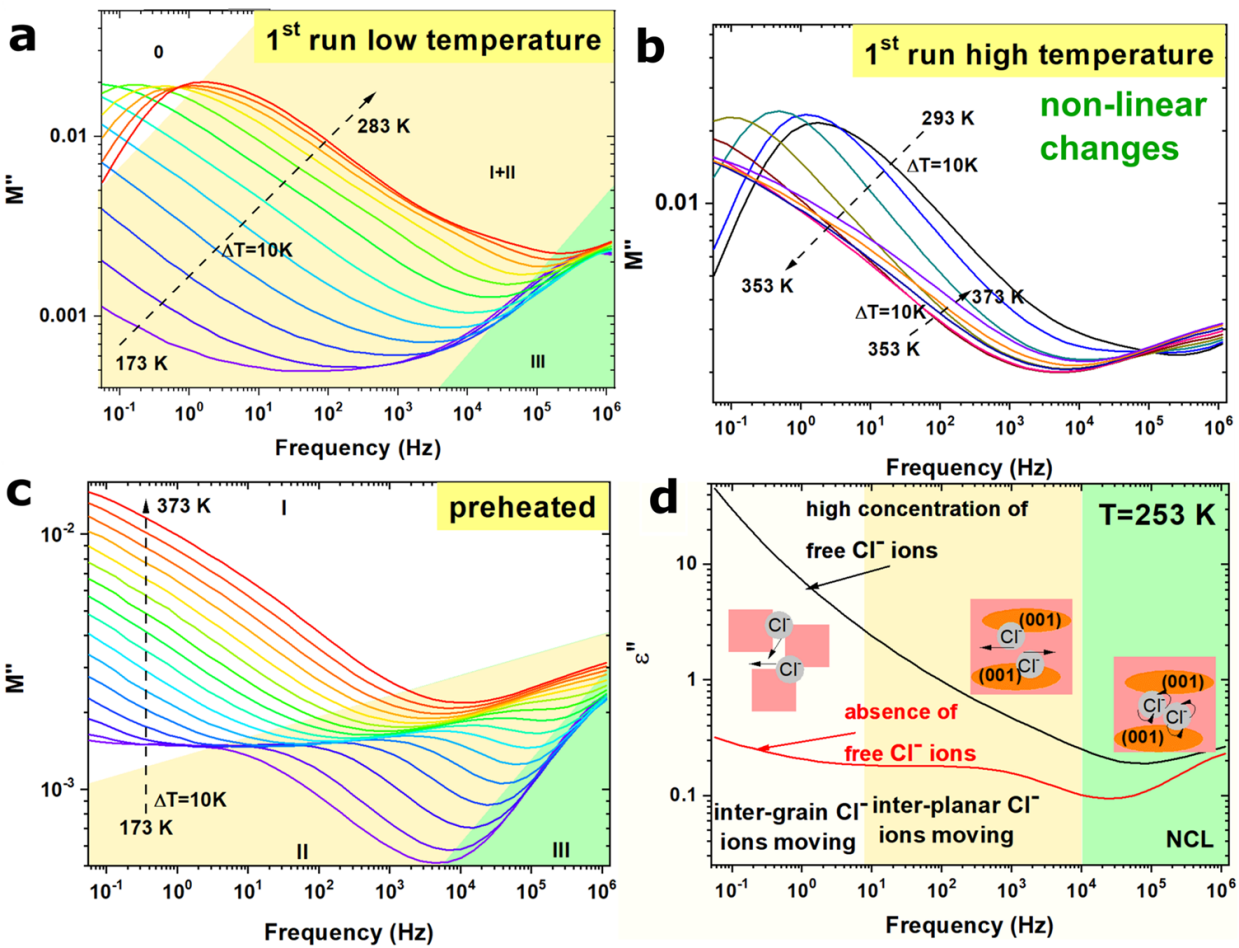
(**a**) Imaginary part of electric modulus *M*″ for BiOCl before heating at 283 K with marked characteristic regions; (**b**) imaginary part of electric modulus *M*″ for BiOCl before heating at high temperatures 293–373 K; (**c**) imaginary part of electric modulus *M*″ for preheated BiOCl over a wide temperature range to 373 K with marked characteristic regions; (**d**) ε″ spectra for BiOCl before (black line) and after heating (red line) with marked regions corresponding to the regions marked in the *M*″ spectra.

In the low-temperature region (173–283 K), characteristics of different nonhomogeneous material relationships can be observed: as the temperature increased, the right shoulder of peak *M″* shifted to higher frequencies, and the maximum peak was observed at 233 K at 10^–1^ Hz (Fig. 4a). This peak was previously associated with the transition between long and short-range mobility. Long-range mobility is associated with grain boundaries, whereas short-range mobility is associated with grains; however, the position of this peak differs from other oxides, such as ferrites. The presence of peak *M″* at low-frequencies below 1 Hz (region 0) was related to the different conduction mechanisms of oxide-based materials. For oxides and similar materials, the conductivity is related to electron movement, whose movement at grain boundaries occurs even at higher frequencies (sometimes even in the kHz region).

The much higher relaxation time expressed as *1/f*_*peak*_ observed for the BiOCl plates is likely related to the fact that the conductivity of this material does not originate from electrons, but rather from chloride ion movements. These ions are large (167 pm ionic radius); therefore, their movement along grain boundaries is very slow. When the frequency exceeds the maximum of *M″*, Cl^−^ movement is impossible, and only their slow movement in the crystal structure (between [Bi_2_O_2_]^2+^ layers) was observed (region I + II); however, especially at low temperatures, a second *M″* peak was observed (Fig. 4a). As can be seen in Fig. 4a,c, this peak was observed for BiOCl before and after heat treatment (region III). This high-frequency electrical process may be related to the presence of a near-constant loss originating from the vibrations of Cl^−^ ions.

Analysis of ε′ showed that when structure rebuilding occurred, the level of free Cl^−^ ions decreased. The same behavior was observed in the *M″* (f) plots (Fig. 4b). The *M″* peak shifts to lower frequencies at higher temperatures (293–353 K), and then a second shift to higher frequencies was observed (353–373 K). Moreover, the *M″* peak disappeared, which proves that there is no transition between long and short-range mobility in the analyzed frequency region. This nonlinear behavior was not observed in the preheated structure. For the stable form of BiOCl, the level of *M″* increased with temperature, and three different regions in the *M″* spectra were marked. The first one (I) is related to the accumulation of Cl^−^ ions at grain boundaries and at the interface between the sample and copper plate. The second region (II) was related to the electrical process related to interplanar Cl^−^ ion movement, which was also observed in the ε*″* spectra (Fig. 4d, red line). As expected, upon increasing the temperature, the chloride ion movement increases and dominates the second process in the higher frequency region. Vibrations of chloride ions and/or polarization of structural defects can also be observed in region III.

This process occurs even at high temperatures. According to this, the interplanar movement of Cl^−^ ions at higher frequencies disappears. The same processes were observed for the ε*″* spectra at constant temperature and in the materials before and after heating. The value is very high at low frequencies and was associated with Maxwell–Wagner polarization, in which free Cl^−^ ions accumulate at grain boundaries. After heating, this process disappears in the same sample, showing that structure rebuilding occurs. A second peak appeared in the *M″* spectra of the preheated sample, which shifted to a higher frequency upon increasing the temperature and peak visible at ε*″* spectra were associated with the reorientation of ions (Cl^−^ ions BiOCl). This has been observed for other crystalline ionic materials such as KH_2_PO_4_ and CsHSeO_4_^33,34^ (region II in Fig. 4c). Moreover, this peak not only shifted to higher temperatures but also disappeared with increasing temperatures, and only peak III appeared in the high-temperature region. This peak was not visible for BiOCl before structure rebuilding because the electric processes were dominated by the movements of free chloride ions. A third, rapid process also occurred at high frequencies and low temperatures and was correlated with the presence of oxygen vacancies in BiOCl; therefore, under heating, no additional changes occur during this relaxation process. It was previously reported that BiOCl rich in oxygen vacancies can be synthesized, and the presence of these defects increased the density of states in the valence band maximum^35^. These oxygen vacancies generate defect dipoles, and the relaxation of these dipoles is visible in the ε*″* (f) spectrum.

### Electrical conductivity

The complex electrical conductivity (σ*) (Eqs. 2 and 3) was determined for BiOCl before and after heat treatment.

2 $$ \sigma^{*} = \omega \varepsilon^{{\prime \prime }} \varepsilon_{0} + i\omega \varepsilon^{{\prime }} \varepsilon_{0} $$

3 $$ \sigma^{{\prime }} = \omega \varepsilon^{{\prime \prime }} \varepsilon_{0} $$

**Figure 5:**
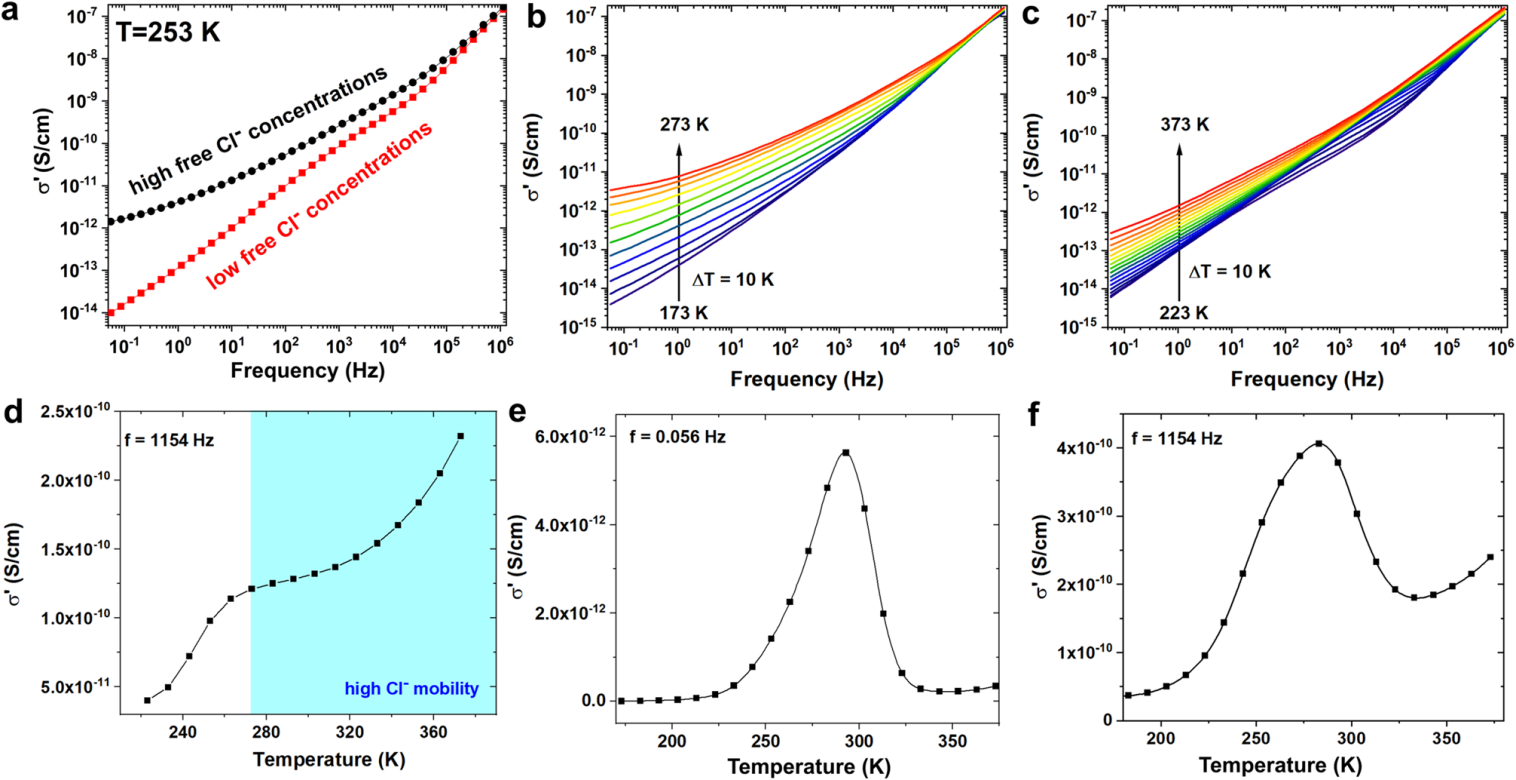
(**a**) Real part of complex conductivity measured for BiOCl before (black points) and after preheating (red points) at 253 K; the influence of temperature on the real part of complex conductivity for BiOCl before (**b**) and after preheating (**c**); (**d**) σ′ (T) relationship measured at 1154 Hz for BiOCl after preheating; σ′ (T) relationship at 0.056 Hz (**e**) and 1154 Hz (**f**) for BiOCl before preheating.

According to the analysis, the low-frequency conductivity of as-synthessized and compressed BiOCl plates is likely dominated by the motion of free Cl^−^ ions. After structure rebuilding, the movement of these ions will be limited, and the conductivity mechanism will change. To describe these changes, the real part of electrical conductivity was measured at 253 K, and both cases are presented in Fig. 5a. The conductivity depends on both the frequency and free Cl^−^ ion concentration. The real part of complex electrical conductivity is nearly 100-times higher for the BiOCl structure rich in free Cl^−^ ions (σ′ = 1.4 × 10^–12^ S/cm at 0.05 Hz) than for the material after rebuilding (σ′ = 9.9 × 10^–15^ S/cm at 0.05 Hz). The difference between the real part of complex conductivity decreases with increasing frequency. As discussed above, the low-frequency dielectric properties, and also the complex conductivity, are related to chloride ion movement. As the frequency increases, the time for this process is too short; therefore, only interplanar ion movement occurs, and conductivity is related only with electron movement. The interplanar movement of chloride ions most likely allows correlated barrier hopping of electrons between D^+^ and D^−^ sites. The high-frequency conductivity (near 10^5^ Hz) may be related to electron tunneling or conductivity by holes generated by oxygen vacancies because the Cl^−^ ion movement is impossible in this frequency range, and they can only vibrate. Therefore, the real part of complex conductivity is much higher than in the low-frequency region.

To confirm this, the influence of temperature on the electrical conductivity was determined, and the measured spectra are presented in Fig. 5b,c. As can be seen in Fig. 5b, the conductivity increases with increasing temperature; however, the temperature did not appear to influence the real part of electrical conductivity for frequencies near 1 MHz. This behavior may be related to the temperature-dependent chloride ion movement in the low-frequency region and temperature non-dependent electrical conductivity by holes or by electron tunneling in the high-frequency region.

The possibility of conduction by holes was confirmed by analyzing the *M″* spectra, in which a high-frequency electrical modulus peak (marked in region III) may be related to the relaxation of defect dipoles generated by oxygen vacancies. It was previously reported that oxygen vacancies and their defect state provide hopping channels and may be responsible for hole conduction^36^. The same high-frequency behavior was observed for BiOCl after heat treatment; however, a much lower electrical conductivity was observed even at higher temperatures. Moreover, as can be seen in Fig. 5c, the conductivity increases with increasing temperature; however, for frequencies above 10 Hz, the changes were different than at low frequencies. This unusual behavior is presented in Fig. 5d as σ′ (T) function for the preheated sample. Two different areas were selected: above and below 273 K. First, conductivity increases very rapidly with increasing temperature, followed by a slowdown in this increase and another rapid increase at very high temperatures. This behavior may be related to interplanar Cl^−^ ion movement. The movement of these ions was significantly limited below 273 K, but it increased above this temperature. On the basis of the performed analysis, it can be concluded, that in the low-temperature region the movement of chloride ions can appear only in the crystallites, whereas above the 273 K the mobility of Cl^−^ ion is much higher and the transfer of these ions between crystallites can appear. These changes between short and long-range movements of chloride ions observed in Fig. 5d can be treated as the origin of structure rebuilding. The changes generated by structure rebuilding were also observed in the σ′ (T) spectra and presented for two chosen frequencies in Fig. 5e,f. The conductivity subsequently increases with the increasing temperature up to 293 K (for 0.056 Hz) and up to 283 K (for 1154 Hz). This 10 K difference may be related to the two different conduction mechanisms in the low and high-frequency regions due to Cl^−^ ion movement along grain boundaries and inside the crystalline structure, respectively. Afterward, a rapid decrease in σ′ was observed in both spectra, which was related to structure rebuilding and the temperature-induced movement of chloride ions from the surface of BiOCl plates into the crystal structure. After this process, the conductivity significantly decreased at 0.056 Hz because of the absence of free Cl^−^ ions, which served as charge carriers at this frequency. Much smaller changes were observed at 1154 Hz because at this frequency, the conductivity was related to interplanar Cl^−^ ion movement, which resulted in the correlated barrier hopping of electrons between D^+^ and D^−^ sites. Therefore, the conductivity was the highest when structure rebuilding occurred (increased movement of chloride ions) and did not decrease to the starting level because a higher Cl^−^ ion concentration in the crystal structure increased the probability of electron hopping.

## Conclusions

BiOCl plates were synthesized using chloramine-T as the chloride ion source. Structure rebuilding at 283 K and the introduction of free Cl^−^ ions into the crystal structure was monitored by changes in broadband dielectric properties and confirmed by XRD patterns and TEM images. Before structure rebuilding, free Cl^−^ ions were responsible for Maxwell–Wagner polarization at low frequencies and high electrical conductivity. The observed *M″* behavior confirmed that the electrical conductivity at low frequencies was related to Cl^−^ ion motions, especially free ions (before structure rebuilding) and ions that slowly moved from the crystal structure (after structure rebuilding). After heating above 283 K, structure rebuilding occurred, which decreased the Cl^−^ ion mobility, and thus the conductivity. At higher frequencies, interplanar movement of Cl^−^ ions occurred in the crystal structure, which was responsible for D^+^ to D^−^ hopping of carriers (probably electrons). This process manifested on ε*″* (f) plots and as *M″* right shoulder for BiOCl after heat treatment. In this study, the very fast relaxation process represented by the *M″* peak in the MHz region was associated with the polarization of structural defects (vacancies) and the movement of holes generated in the material. The high-frequency conductivity (near 10^5^ Hz) may be related to electron tunneling or conductivity by holes generated by oxygen vacancies. This is because the Cl^−^ ion movement cannot occur in this frequency range, and they can only vibrate. The presence of highly mobile free chloride ions at temperatures up to 283 K in the compressed sample is important to construction, especially for efficient chloride ion batteries.
